# Quality of Life in Community-Dwelling Older People with Functional and Nutritional Impairment and Depressive Symptoms: A Comparative Cross-Sectional Study in Brazil and Portugal

**DOI:** 10.3390/geriatrics7050096

**Published:** 2022-09-13

**Authors:** Jéssica Maria Arouca de Miranda, Dalyanna Mildred de Oliveira Viana, Anderson Antônio Lima dos Santos, Áquila Filêmon de Andrade Costa, Bruno Araújo da Silva Dantas, Francisco Arnoldo Nunes de Miranda, Felismina Rosa Parreira Mendes, Gilson de Vasconcelos Torres

**Affiliations:** 1 Centro de Ciências da Saúde, Federal University of Rio Grande do Norte (UFRN), Avenida Governador Silvio Pedroza, 306, Areia Preta. Ed Portal da Enseada, ap 100, Natal 59014-100, RN, Brazil; 2 Centro de Ciências da Saúde, Federal University of Rio Grande do Norte (UFRN), Rua Piquia, 7932, Pitimbu, Natal 59067-580, RN, Brazil; 3 Department of Nursing, Federal University of Rio Grande do Norte (UFRN), Rua Cantor Carlos Alexandre, Lagoa Azul, Natal 59138-623, RN, Brazil; 4 Department of Nursing, Federal University of Rio Grande do Norte (UFRN), Rua Cerejeiras, 1914-Potengi, Natal 59120-300, RN, Brazil; 5 Faculdade de Ciências da Saúde do Trairi, Federal University of Rio Grande do Norte (UFRN), Rua Alice Azevedo, 30, Natal 59080-015, RN, Brazil; 6 Departamento de Enfermagem, Federal University of Rio Grande do Norte (UFRN), Av. Ayrton Senna, 10, Capim Macio, Natal 59080-100, RN, Brazil; 7 Escola de Enfermagem São João de Deus, University of Évora, Largo dos Colegiais 2, 7004-516 Évora, Portugal; 8 Centro de Ciências da Saúde, Federal University of Rio Grande do Norte (UFRN), Rua das Massarandubas, 292, Nova Parnamirim, Parnamirim 59150-630, RN, Brazil

**Keywords:** aged, quality of life, functional status, nutritional assessment, depression

## Abstract

Background: The interaction of quality of life (QoL) with functionality, nutrition and depression has been studied, but few studies have compared different realities. Our objective was to compare the associations of QoL with impaired functionality, nutritional status and depressive symptoms among older people patients treated in primary health care (PHC) in Brazil and Portugal. Methods: Cross-sectional, comparative study was conducted with primary data from PHC services in Brazil and Portugal with users over 65 years old. Participants’ scores were classified as “impaired” and “preserved” for QoL, functional decline, nutrition and depression. We used Pearson’s chi-square test, Fisher’s exact test and the Mann–Whitney U test. Results: Our sample had a total of 150 PHC users. We found lower QoL scores in Brazil, which were associated with the risk of functional decline for the domains Physical Functioning, General Health Perceptions, Mental Health dimensions and Physical Health. Nutritional impairment in the group from Portugal included the domains of Vitality and Social Role Functioning. For depressive impairment, Portugal showed an association with the domains Mental Health, Vitality and Social Role Functioning. Conclusions: QoL was associated with functional and nutritional impairment and depressive symptoms, highlighting physical, mental and social characteristics related to the perception of well-being.

## 1. Introduction

The World Health Organization (WHO) defines quality of life (QoL) as an individual’s perception of his or her roles, values, culture, goals, expectations and concerns [1]. In the context of older people, QoL entails the interaction between various aspects involving their autonomy, participation in society and basic needs linked to functionality, nutrition and mental health [2].

Functionality is an important component in the QoL of older people, as its decline implies a loss of autonomy [3]. Poor functionality impairs the performance of basic activities of daily living (ADL), which include adequate preparation and consumption of food and activities that promote physical and mental well-being [4].

Poor nutrition is one of several important health and QoL problems experienced by older people, which can cause functional and mental declines and result from such declines [5]. It is a common syndrome in the older people and refers to insufficient dietary intake, lack of appetite, loss of muscle mass and unintentional weight loss and can lead to serious outcomes, especially in this age group, such as frailty and sarcopenia [6,7].

Among the older population, depression is common and affects cognition, emotion, behavior, functionality and QoL, culminating in high rates of recurring depressive symptoms and an inability to perform occupational and social activities [8].

Functional impairments, poor nutrition and depressive symptoms in older people have been studied within different contexts, leading to the belief that these factors may have an impact on QoL [3,6]. However, whether these factors and consequences are different in countries with different socioeconomic realities, such as Brazil and Portugal, is worth questioning. Primary health care (PHC) in Brazil acts to promote health and prevent diseases [9]. However, physical and mental illnesses are often present with hospitalizations, which is attributed to failures in the process of welcoming and tracking patients with these demands for care services [10]. The National Health Survey conducted in 2019 showed that 10.2% of Brazilian individuals were diagnosed with depression, most of whom were older people. More than 9% had some functional limitations related to ADL, and only 17% consumed an adequate amount of fruits and vegetables in their diets [11].

Portugal is a country that resembles Brazil in relation to the epidemiological context of physical and mental diseases but presents better evaluations of its PHC, especially in its accessibility and success in prevention and screening actions [12]. The National Health Survey of 2019 showed a progressive increase in chronic diseases related to physical and mental health, such as hypertension, diabetes mellitus, low back pain and depression, compared to the previous five years. Among older people, functional dependence was found in at least 13% needing assistance with some ADLs, such as bathing, getting out of bed or dressing, and this percentage was above the average found throughout the European Union [13].

Despite the availability of these findings, new knowledge about the different scenarios in which older people have physical, nutritional and depressive disorders raises questions about how these impairments affect their QoL and the role that cultural factors might play. Filling this scientific gap implies providing support for the construction of new PHC policies and implementing assertive measures in the prevention of health problems and QoL preservation. PHC is also understood to be the level of care that has the greatest potential for effective prevention of diseases and injuries, especially in the older population, which is considered one of the most vulnerable populations in this context. Therefore, we conducted this comparative study between Brazil and Portugal to elucidate the questions that emerge in the face of these demands.

The primary objective of this study was to compare the associations of QoL with impaired functionality, nutritional status and depressive symptoms among older patients seen in PHC in Brazil and Portugal.

## 2. Materials and Methods

### 2.1. Design

A cross-sectional, comparative study with primary data occurred in the PHC services of Brazil and Portugal from 2017 to 2018. Our hypothesis was that the levels of association between QoL and impaired functionality, nutritional status and depressive symptoms differ among older people Brazilian and Portuguese individuals treated through PHC.

### 2.2. Ethical Aspects

The study was subjected to ethical review by the Research Ethics Committee of the Onofre Lopes University Hospital (CEP/HUOL) in Brazil, with approval no. 562318. In Portugal, the study was evaluated and approved by the CEP of the University of Évora (no. 14011) and the Ethics Committee for research in the areas of human health and well-being at the University of Évora (no. 17006/2018). The study followed the principles of good practice in medical research considering the Declaration of Helsinki during its development.

Each participant was presented with an informed consent form with guidance on the objective, importance, risks and benefits of the study. The signatures of all participants were collected before the beginning of each interview.

### 2.3. Study Location

In Brazil, the study took place in the cities of Natal and Santa Cruz, state of Rio Grande do Norte, in regional PHC units and in spaces belonging to the Federal University of Rio Grande do Norte (RN). In Portugal, older people care services are linked to the regional health of the Council of Évora city and belong to the National Health System of Portugal.

### 2.4. Population and Sample

The study population was Brazilian and Portuguese older people using PHC services. For both Brazilian and Portuguese individuals, the age criterion was 65 years or older, defined according to the criteria established by the WHO [14]. In addition to the cutoff age, for inclusion in the study, participants should be registered in the PHC service of their research setting (Brazil/Portugal) at least 6 months before completion of the study and present a preserved cognitive state, as confirmed by the Mini-Mental State Examination (MMSE), with a minimum score of 17 points [15]. The following exclusion criteria were adopted: permanent or transient physical disability at the time of data collection and reports of personal or family trauma in a period less than or equal to 6 months prior to the time of the study.

We obtained a nonprobabilistic sample (selected by convenience). The sample size was calculated individually for each scenario. In Brazil, the total older population treated in two PHC units (one in Natal and another in Santa Cruz) was used for sample size calculation, and we estimated that 135 people were needed. In Portugal, we targeted older patients seen in a PHC unit of the Council of Évora and estimated that 70 patients were needed. With a 95% confidence interval and 5% margin of error, our calculations resulted in a total of *n* = 100 in Brazil and *n* = 60 in Portugal; this result was obtained with the aid of an online calculator accessible at https://calculareconverter.com.br/calculos-amostral (accessed on 15 June 2022). A total of 150 people completed the study (Brazil *n* = 100 and Portugal *n* = 50).

The Brazilian participants were the first interviewees and served as a baseline for the study and its socioeconomic characterization as a reference for pairing with the group from Portugal. Thus, to eliminate possible confounding factors generated by the differences between the two groups, the socioeconomic profiles of the Brazilian individuals were grouped using the following variables in a dichotomous structure: sex (male/female), age group (65–80/81–100 years), marital status (with partner/without partner), family income in minimum wages (up to 1/>1) and chronic diseases (presence/absence). Each combination of these five variables resulted in a code that was then used to label the participants. That is, each individual with the same combination of characteristics was represented by one of these codes. For each code, we established that at least one individual from each group (Brazil/Portugal) should fulfill the associated criteria, and those that did not meet this criterion were excluded.

### 2.5. Data Collection and Availability

Data were collected in face-to-face interviews by undergraduate and graduate students in nursing and nutrition. No remuneration, incentives or blinding of any of those involved in the study was applied.

To prevent errors in the interpretation of the content of the instruments by the interviewers, successive training sessions were conducted with the team of researchers involved, prior to the data collection, addressing cultural aspects relevant to the countries during the training sessions.

The interviews took place on scheduled days, at the PHC services or at the participants’ homes, with the presence of health professionals from those coverage units and a duration of approximately one hour for each of them. In Brazil, they were performed from December 2017 to March 2018, and in Portugal, they were performed during the month of July 2018. During data collection, a researcher was responsible for supervision to guarantee the quality of the interviews and the reliability of the information.

### 2.6. Instruments and Variables

In the socioeconomic characterization of the sample, the questionnaire asked for socioeconomic data composed of categorical variables regarding sex, age, education, marital status and income.

To evaluate QoL, we used the Medical Outcomes Short-Form Health Survey QoL (SF-36), which evaluates 10 variables based on 36 questions on a Likert scale. The survey generates a total score and addresses domains and dimensions of physical health, functional, emotional, social, pain, vitality and general health status. The evaluation of each variable generates a score of 0–100 points in ascending order from the worst to the best QoL. Each of the domains and dimensions are represented by a group of quiz questions. The answers to each question are graded on a Likert scale. The calculation for each domain and dimension is the result of the score generated by its respective group of questions. [16].

The assessment of the risk of functional decline was performed using the Prisma 7 (Quebec, CA) instrument, which consists of seven dichotomous questions (yes/no) that indicate the presence of risk in at least three positive responses [17]. The Lawton & Brody Scale was used to evaluate functionality in the context of Instrumental Activities of Daily Living (IADL), such as locomotion and transportation, handling of money and autonomy to use medications and perform simple household activities, with a maximum score of 27 points and classification proportional to the number of points for “total dependence”, “partial dependence” or “independent” [18].

For the nutritional assessment, we used the Mini Nutritional Assessment (MAN), which is a screening test and a global assessment. Body mass index (BMI), weight loss in the last three months, physical mobility, housing conditions and medication use are measured, and the results classify individuals as “malnourished”, “at risk of malnutrition” or “eutrophic”. This instrument generates a maximum score of 24 points, indicating the best possible nutrition [19,20].

To evaluate the presence and levels of depressive symptoms, we used the Geriatric Depression Scale, version with 15 questions (GDS-15), which aims to investigate the presence of these symptoms in older people. Responses to the questions are dichotomous (yes/no), classifying the levels of depressive symptoms as “absent”, “mild”, “moderate” or “severe” with a maximum score of 15 points, which corresponds to its worst evaluation [21,22]. All scales used were translated and validated for Portuguese, the official language in both study locations.

The categorical variables of each of the scales were regrouped to show the presence or absence of impairment. Thus, the classifications of “total dependence” and “partial dependence” (IADLs, Lawton & Brody) were considered “impaired”; “risk of functional decline” (Prisma 7); “malnourished” and “at risk of malnutrition” (nutritional, MNA); and “mild”, “moderate” and “severe” (Depression-GDS-15). The other categories of the scales were reclassified as “preserved”, indicating no impairment in the evaluated variable. This procedure was performed to obtain the subcategory of impaired individuals and evaluate their association with QoL.

### 2.7. Data Analysis and Treatment

The data were tabulated and organized into tables and banks using Microsoft Excel 2016 (Microsoft Corporation, Washington, WA, USA). To perform the statistical analysis, we used the Statistical Package for the Social Sciences, version 20.0 (International Business Machines Corporation, Armonk, NY, USA), for the normality test (Kolmolgorov-Smirnov), which indicated the nonnormality of the independent variables of QoL. Thus, nonparametric tests, Pearson’s chi-square association or Fisher’s exact test were used for categorical variables of socioeconomic characterization, functional decline, functionality, nutrition and depressive symptoms between the study groups. The Mann–Whitney U test, which was used for scalar variables of QoL in the comparative analyses between the groups from Brazil and Portugal, presented as percentiles (25, 50 and 75) in the scores of the QoL domains (complete sample), as well as for their crosses with the scales of functional decline, poor nutrition and depressive symptoms, in the cutoff for the compromised individuals. We adopted a confidence interval level of 95% and *p* value ≤ 0.05 as criteria for statistical significance [23].

## 3. Results

**Figure 1:**
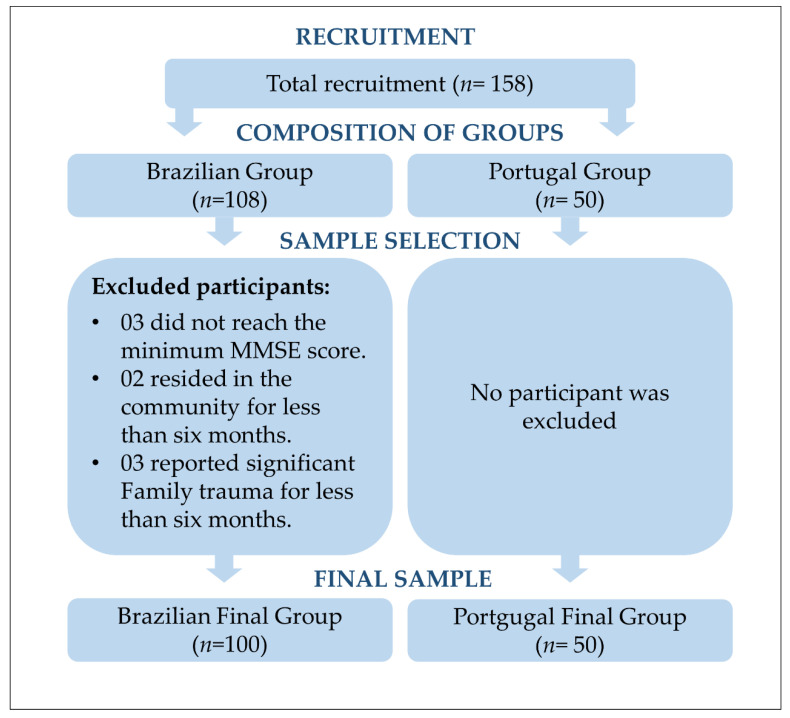
Flowchart of the recruitment and selection process for participants according to the exclusion criteria.

Our sample had a total of 150 PHC users (100 Brazilian and 50 Portuguese individuals). Obeying the inclusion and exclusion criteria established for the study, some of the Brazilian participants were excluded, and none were discarded in Portugal, as shown in Figure 1.

Table 1 describes the socioeconomic characterization of the sample, which shows a predominance of females in both Brazil (73%) and Portugal (82%), and the age group 65–80 accounted for 89% and 86% of the sample, respectively. The mean age of the total sample was 74.0 years (standard deviation [SD] = 6.61). The average age was 73.3 years in Brazil (SD = 5.67) and 75.3 years in Portugal (SD = 8.08).

Despite the pairing performed between the two groups, we observed significant differences between them in the education variable. More than half of both groups had less than five years of education, which was observed in 79% of Brazilian and 52% of Portuguese individuals (*p* value = 0.001). We also observed significance in the income variable, with a higher percentage of individuals receiving less than minimum wage in Brazil (42%) and 100% in Portugal (*p* value < 0.001).

Table 2 shows the association of the scores for the domains and QoL dimensions among the participants per group, which are described by their percentiles. In general, the group from Portugal showed a trend of higher scores in most domains, with statistical significance in Physical Functioning (*p* value < 0.001), General Health Perceptions (*p* value = 0.043) and Social Role Functioning (*p* value = 0.002), which impacted the total score (*p* value = 0.006). Additionally, with higher scores for Portugal, the Physical Health dimension stood out (*p* value < 0.001).

Table 3 shows the analysis of the associations of the status (impaired/preserved) of the variables evaluated between the research scenarios. When evaluating the risk of functional decline (Prisma 7), we observed that the most impaired group was the group from Portugal (82%), with Brazilian individuals presenting a lower proportion (56%) (*p* value = 0.002). However, 75% of Brazilian individuals showed impairments in functionality in the context of IADLs (Lawton & Brody) (*p* value < 0.001).

In the nutritional and depressive symptom assessments, the Brazilian group showed a higher percentage of impairment in both variables, with the MNA scale indicating that 90% of this group was poorly evaluated (*p* value < 0.001).

To better represent the behavior of the association between the QoL domains and each of the evaluated variables and their respective scales, we constructed the following tables for each of them, considering only individuals with “impaired” status.

Table 4 shows that among the individuals with impairment in functional decline (Prisma 7) (*n* = 97), the group from Brazil showed a tendency for lower scores compared to Portugal in almost all variables, highlighting the domains of Physical Functioning (*p* value = 0.006), General Health Perceptions (*p* value = 0.042), Mental Health (*p* value = 0.042) and Physical Health (*p* value = 0.031).

The behavior of IADLs (Lawton & Brody) between the two groups showed a trend, without statistical significance, of lower scores in Brazil for most variables of QoL. Due to the incipience of the representativeness of these results, we decided not to present the data in a table.

When evaluating individuals with impaired nutritional status (*n* = 122), we found that, for this variable, the group from Portugal obtained lower scores for most variables of QoL, with emphasis on the domains of Vitality (*p* value = 0.049) and Social Role Functioning (*p* value = 0.013). Brazil, in turn, had significantly lower scores in the Physical Functioning (*p* value = 0.006) and Physical Health domains (*p* value = 0.032).

In terms of the QoL among participants with impaired levels of depressive symptoms (GDS-15) (*n* = 68), we found that overall, Brazilian and Portuguese individuals had lower scores for similar numbers of variables. However, the Portuguese group exhibited this behavior significantly in the domains of Mental Health (*p* value = 0.016), Vitality (*p* value = 0.050) and Social Role Functioning (*p* value = 0.016), while Brazilian individuals scored higher in the General Health Perceptions domain (*p* value = 0.041).

## 4. Discussion

Our study showed that when comparing the two groups, we observed greater impairment of functionality, poor nutrition and depressive symptoms in the Brazilian group. The risk of functional decline and nutrition showed an association, especially with the physical components of QoL and those that imply the perception of well-being. The group from Portugal, in turn, showed an association between poor nutrition and depressive symptoms with the domains Vitality, Social Role Functioning and Mental Health. These data present an important and unprecedented situational diagnosis of the two countries, which have different cultures and levels of PHC structure. Understanding the reality of different scenarios raises the need for the construction of new public health policies for the older population with a preventive focus on the functional, nutritional and depressive symptoms components [6,24,25].

When establishing the profile of the participants, we observed a predominance of female older people and younger age groups, data that are similar to those of other studies [26,27]. However, although we applied rigorous sample selection criteria for the pairing of the groups according to five socioeconomic variables, we found significant differences between the participants from Brazil and Portugal. The level of education and income stood out in this perspective. Even though education was not a criterion for pairing the groups, whether inequities in education and income could influence our results is important to question, since socioeconomic factors have been shown to mediate QoL outcomes, functionality, nutrition and depressive symptoms [6,24,25,28,29].

Regarding functionality, the group from Portugal showed greater impairment when measuring the risk of functional decline (Prisma 7). However, we found controversial data that show the status of functionality with predominant impairment among Brazilian individuals according to the Lawton & Brody scale. This finding about the Brazilian group is similar to that found in a study conducted in Spain, which indicated functional impairment in most of the individuals studied; however, these were older people who required caregivers [30]. This situation requires more dependence than that of the individuals in our sample and suggests that the Brazilian individuals surveyed have functional statuses similar to those dependent on caregiver assistance.

Given the analysis of individuals with impairment, Brazilian individuals showed associations between the domains of Physical Functioning and General Health Perceptions and the risk of functional decline, with lower scores compared to the Portuguese group. When analyzing the status of functionality (IADLs), Brazilian subjects were observed to have lower scores for almost all variables, with the domains Physical Functioning, General Health Perceptions, Mental Health and Physical Health standing out. In both China [31] and Thailand [32], the authors found similar evidence, which indicated an association between functional impairment and physical appearance, in addition to the mental component. This association may be because the functional decline observed during aging implies impairment in the ability to perform ADL, with psychological, physical and social ramifications [33]. Alternatively, the association could be the result of the impact that nutritional status has on functional dependence and cognitive impairment [34], since we also found changes in nutrition in our sample.

We observed that the group in Brazil showed greater impairment in nutrition, along with depressive symptoms. Among the individuals with nutritional impairment, Brazilian participants obtained lower QoL scores than Portuguese, showing an association with the Physical Functioning and Physical Health domains. Among the subjects with depressive symptoms, we found that those from Portugal had worse scores associated with the domains of mental health, Vitality and Social Role Functioning. This behavior between the groups can be explained by the negative impact that malnutrition has on all domains and dimensions of QoL [35]. In addition, data indicate that nutrition has a greater impact on QoL than depressive symptoms [36].

However, the results found in Canada [36], Spain [37], China [28] and Ethiopia [8] can be considered valid, which show that depression is a strong predictor of worse QoL scores. In our study, we observed results that point to a similar association, with emphasis on subjects from Portugal who showed greater depressive symptoms. They obtained lower scores in the Mental health, Vitality and Social Role Functioning domains, while in Brazil, this association was present in the General Health Perceptions domain. Among these findings, the relevance of the social component stands out, which was pointed out in a study conducted in Vietnam as one of the main factors associated with depression and is related to the other variables, since social interaction is assumed to be a protective factor for mental health and QoL [26].

Notably, the social variable is strongly influenced by collective activities, as well as coexistence with other individuals. In recent years, interventions with group activities and alternatives and adjuvants to pharmacological treatments for the preservation and improvement of various aspects of QoL have been tested. Different strategies have been tested with PHC in this regard. Among them, one strategy that was applied in Brazil was a multidimensional approach: education, incentives for physical exercise, adequate diet and social interaction with the use of technological resources, which had positive outcomes for depressive symptom and QoL improvements [38]. In Singapore, improvement of these variables was also observed by augmenting the usual PHC care with specialized care for depressive symptoms using educational and nonpharmacological resources [39].

These interventions help with some aspects of the well-being of individuals and psychological and physical elements that affect QoL [40,41]. Our findings raise a concern that should be considered in the development of public health policies because the impairment of functional and nutritional variables and depressive symptoms have negative effects on each other. That is, the individual with one of these compromised elements tends to show losses in other areas, either in the short or long term [42,43]. In summary, our study showed that aspects of QoL were associated in a reasonably different way in the affected individuals of the sample, depending on the scenario studied.

Therefore, notably, Brazil and Portugal have very different socioeconomic and cultural characteristics, which also affect the quality of care in their respective PHC facilities [44]. Health services in Brazil have received poor evaluations in most regions, especially in their specialized care network. The coverage of Brazilian PHC services has as a prerogative close contact with individuals in the community to establish a bond of trust between the population and the service team [9]. However, monitoring of its users regarding adherence to health treatments is impaired, often motivated by the stigma related to health problems, which requires that this bond be well established [45].

Portugal, in turn, has a better evaluation history, especially with regard to user satisfaction with service accessibility [12,46]. PHC in both countries plays an important role in the prevention and monitoring of health problems. The countries’ services are similar in that they are focused on the prevention and treatment of chronic diseases, such as hypertension and diabetes, and such services are often reduced to quick consultations without in-depth investigations or with mere distribution of medications [47,48]. These differences are among our findings, and we found significance in relation to income and educational level between the countries. However, we found impairment status in a considerable part of the sample from both countries.

### Limitations

As a limitation of our study, we cite the small overall sample size and the imbalanced sample sizes between Brazil and Portugal. In the Portugal scenario, we were unable to reach the quantitative value obtained in our sample calculation. Additionally, no blinding of the participants and researchers was performed. Another important fact was that we did not investigate whether the individuals had already received any follow-up or specialized treatment in parallel with the service offered by the PHC in their community, which we believe could influence our findings. Thus, a causal relationship cannot be established with the external validity of the data found. The results compare the situation of two very different countries at cultural and social levels and with different health systems, which may have hampered the power of the comparison between them. Additionally, the cross-sectional design also contributes to the lack of inferences. Despite the caution adopted during data collection, convenience sampling may indicate a bias in the study due to the absence of the probabilistic method. To mitigate possible biases and confounding factors, we paired subjects from the two countries according to their socioeconomic profiles to promote similarity between them. In addition, we excluded individuals who had potential physical or mental health impairments that could presumably impact their QoL or other variables evaluated.

## 5. Conclusions

The QoL of our older people subjects was associated with functional and nutritional impairment and depressive symptoms, especially when aspects of their general well-being (physical, mental and social) were evaluated. In the comparison between the affected individuals in the studied groups, we observed that the group from Brazil had lower scores in the functional and nutritional assessments, while the group from Portugal had lower scores for depressive symptoms. Thus, our evidence confirmed that our hypothesis was supported.

Given these results, we suggest a clinical trial with thorough planning of effective interventions for each of the variables mentioned to improve their performance or attenuate their progression with the aging process.

## Figures and Tables

**Table 1:** Socioeconomic characteristics between Brazil and Portugal.

Variable		Brazil (*n* = 100) *n* (%)	Portugal (*n* = 50) *n* (%)	Total (*n* = 150) *n* (%)	*p* Value *
Sex	Female	73 (73%)	41 (82%)	114 (76%)	0.224
	Male	27 (27%)	9 (18%)	36 (24%)	
Age range, yr	65–80	89 (89%)	43 (86%)	132 (88%)	0.594
	81–100	11 (11%)	7 (14%)	18 (12%)	
Educational attainment, yr	≤5	79 (79%)	26 (52%)	105 (70%)	0.001
	>5	21 (21%)	24 (48%)	45 (30%)	
Marital status	Married/cohabitating	51 (51%)	22 (44%)	73 (48.7%)	0.419
	Single/widowed/divorced	49 (49%)	28 (56%)	77 (51.3%)	
Household income, ** minimum wage	≤1	42 (42%)	50 (100%)	92 (61.3%)	<0.001
	>1	58 (58%)	-	58 (38.7%)	
Chronic Diseases	Yes	79 (79%)	46 (92%)	125 (83.3%)	0.062 ***
	No	21 (21%)	4 (8%)	25 (16.7%)	

yr: years; ≤, less than or equal to; >, greater than; * Pearson’s Chi-Squared test; ** Minimum wage, R$ 954.00 (BRL) in Brazil/€618.00 (EUR) in Portugal (2018); *** Fisher exact test.

**Table 2:** Quality of life differences between Brazil and Portugal.

QoL (SF-36)	Brazil (*n* = 100)	Portugal (*n* = 50)	*p* Value *
	Percentiles	Percentiles	
	25-50-75	25-50-75	
Domains			
Physical role Functioning	35.0–67.5–90.0	50.0–75.0–90.0	0.174
Physical Functioning	0–50–100	75–100–100	<0.001
Emotional Role Functioning	33.3–100–100	66.7–100–100	0.359
Mental Health	52–56–60	48–56–64	0.332
General Health Perceptions	30–35–55	35–50–56.2	0.043
Pain	20–40–60	20–30–40	0.076
Vitality	45–50–60	43.7–50–56.2	0.348
Social Role Functioning	50–50–50	37.5–50–50	0.022
Total Score	44.2–55–61	53.9–60.8–63.9	0.006
Dimensions			
Mental Health	47.2–57–61	49.9–58.3–61.9	0.235
Physical Health	40–50–57	49.7–57–63	<0.001

QoL: quality of life; * Mann–Whitney U test; *p* values refer to the differences in QoL and subdomains between Brazil and Portugal.

**Table 3:** Associations of the evaluated variables between Brazil and Portugal.

Evaluated Variables	Rating	Brazil (*n* = 100)	Portugal (*n* = 50)	Total (*n* = 150)	*p* Value *
Functionality					
Prisma 7	I **	56 (56%)	41 (82%)	97 (64.7%)	0.002
	P ***	44 (44%)	9 (18%)	53 (35.3%)	
Lawton & Brody	I	75 (75%)	12 (24%)	87 (58%)	<0.001
	P	25 (25%)	38 (76%)	63 (42%)	
Nutrition					
MNA	I	90 (90%)	32 (64%)	122 (81.3%)	<0.001
	P	10 (10%)	18 (36%)	28 (18.7%)	
Depressive Symptoms					
GDS-15	I	59 (59%)	19 (38%)	78 (52%)	0.015
	P	41 (41%)	31 (62%)	72 (48%)	

MNA: Mini Nutritional Assessment; GDS-15, Geriatric Depression Scale; * Pearson’s Chi-Squared test; ** Impaired; *** Preserved. Criteria for Impaired Status: “risk of functional decline” (Prisma 7); “total dependence” and “partial dependence” (IADLs, Lawton & Brody); “malnourished” and “at risk of malnutrition” (nutritional, MNA); “mild”, “moderate” and “severe” (Depression-GDS-15). Criteria for preserved status: No problem.

**Table 4:** Association between QoL and impairment for the variables evaluated.

QoL (SF-36)	Risk of Functional Decline (Prisma 7)		Nutrition (MNA)		Depressive Symptoms (GDS-15)	
	Brazil (*n* = 56)	Portugal (*n* = 41)	Brazil (*n* = 90)	Portugal (*n* = 32)	Brazil (*n* = 59)	Portugal (*n* = 19)
	Percentiles		Percentiles		Percentiles	
	25–50–75	25–50–75	25–50–75	25–50–75	25–50–75	25–50–75
Domains						
Physical Functioning	50–100–100	100–100–100	0–50–100	37.5–100–100	0–25–100	25–100–100
*p* value *	0.006		0.006		0.054	
Mental Health	56–58–60	50–60–64	52–56–60	45–52–60	52–56–60	40–48–56
*p* value	0.677		0.098		0.016	
General Health Perceptions	30–35–50	30–45–55	30–37–55	36.2–50–60	30–40–60	40–50–65
			0.093			
*p* value	0.042				0.041	
Vitality	45–50–60	45–50–60	45–50–60	40–45–55	45–50–60	40–45–50
*p* value	0.827		0.049		0.050	
Social Role Functioning	50–50–50	43.7–50–50	50–50–53	37–50–50	50–50–62	37.5–50–50
*p* value	0.072		0.013		0.016	
Dimensions						
Physical Health	49.2–55–59	52–58–63	39–50–57	45.2–53.5–62.5	38–45–56	43–52–58
*p* value	0.031		0.032		0.147	
Mental Health	49–57–60	51.6–59.9–62.6	45.7–57–61	48.1–57.7–61	43–56–61	40.6–50.2–61.2
*p* value	0.042		0.848		0.608	

Subtitle: MNA, Mini Nutritional Assessment; GDS-15, Geriatric Depression Scale; QoL, quality of life; * Mann–Whitney U test.

## Data Availability

The data presented in this study are openly available in Mendeley Data at DOI 10.17632/zm7b8dd9sj.2. These data can be found here: https://data.mendeley.com/datasets/zm7b8dd9sj/1 (accessed on 15 June 2022).
